# Cost-effectiveness Analysis of Anatomic vs Functional Index Testing in Patients With Low-Risk Stable Chest Pain

**DOI:** 10.1001/jamanetworkopen.2020.28312

**Published:** 2020-12-14

**Authors:** Júlia Karády, Thomas Mayrhofer, Alexander Ivanov, Borek Foldyna, Michael T. Lu, Maros Ferencik, Amit Pursnani, Michael Salerno, James E. Udelson, Daniel B. Mark, Pamela S. Douglas, Udo Hoffmann

**Affiliations:** 1 Cardiovascular Imaging Research Center, Massachusetts General Hospital, Harvard Medical School, Boston; 2 MTA-SE Cardiovascular Imaging Research Group, Heart and Vascular Center, Semmelweis University, Budapest, Hungary; 3 School of Business Studies, Stralsund University of Applied Sciences, Stralsund, Germany; 4 Knight Cardiovascular Institute, Oregon Health and Science University, Portland; 5 Cardiology Division, Evanston Hospital, Evanston, Illinois; 6 Departments of Medicine and Radiology, University of Virginia Health System, Charlottesville; 7 Division of Cardiology, Tufts Medical Center, Boston, Massachusetts; 8 Duke Clinical Research Institute, Duke University School of Medicine, Durham, North Carolina; 9 Division of Cardiology, Department of Medicine, Duke University School of Medicine, Durham, North Carolina

## Abstract

**Importance:**

Both noninvasive anatomic and functional testing strategies are now routinely used as initial workup in patients with low-risk stable chest pain (SCP).

**Objective:**

To determine whether anatomic approaches (ie, coronary computed tomography angiography [CTA] and coronary CTA supplemented with noninvasive fractional flow reserve [FFR_CT_], performed in patients with 30% to 69% stenosis) are cost-effective compared with functional testing for the assessment of low-risk SCP.

**Design, Setting, and Participants:**

This cost-effectiveness analysis used an individual-based Markov microsimulation model for low-risk SCP. The model was developed using patient data from the Prospective Multicenter Imaging Study for Evaluation of Chest Pain (PROMISE) trial. The model was validated by comparing model outcomes with outcomes observed in the PROMISE trial for anatomic (coronary CTA) and functional (stress testing) strategies, including diagnostic test results, referral to invasive coronary angiography (ICA), coronary revascularization, incident major adverse cardiovascular event (MACE), and costs during 60 days and 2 years. The validated model was used to determine whether anatomic approaches are cost-effective over a lifetime compared with functional testing.

**Exposure:**

Choice of index test for evaluation of low-risk SCP.

**Main Outcomes and Measures:**

Downstream ICA and coronary revascularization, MACE (death, nonfatal myocardial infarction), cost, quality-adjusted life-years (QALYs), and incremental cost-effectiveness ratio (ICER) of competing strategies.

**Results:**

The model cohort included 10 003 individual patients (median [interquartile range] age, 60.0 [54.4-65.9] years; 5270 [52.7%] women; 7693 [77.4%] White individuals), who entered the model 100 times. The Markov model accurately estimated the test assignment, results of anatomic and functional index testing, referral to ICA, revascularization, MACE, and costs at 60 days and 2 years compared with observed data in PROMISE (eg, coronary CTA: ICA, 12.2% [95% CI, 10.9%-13.5%] vs 12.3% [95% CI, 12.2%-12.4%]; revascularization, 6.2% [95% CI, 5.5%-6.9%] vs 6.4% [95% CI, 6.3%-6.5%]; functional strategy: ICA, 8.1% [95% CI, 7.4%-8.9%] vs 8.2% [95% CI, 8.1%-8.3%]; revascularization, 3.2% [95% CI, 2.7%-3.7%] vs 3.3% [95% CI, 3.2%-3.4%]; 2-year MACE rates: coronary CTA, 2.1% [95% CI, 1.7%-2.5%] vs 2.3% [95% CI, 2.2%-2.4%]; functional strategy, 2.2% [95% CI, 1.8%-2.6%] vs 2.4% [95% CI, 2.3%-2.4%]). Anatomic approaches led to higher ICA and revascularization rates at 60 days, 2 years, and 5 years compared with functional testing but were more effective in patient selection for ICA (eg, 60-day revascularization-to-ICA ratio, CTA: 53.7% [95% CI, 53.3%-54.0%]; CTA with FFR_CT_: 59.5% [95% CI, 59.2%-59.8%]; functional testing: 40.7% [95% CI, 40.4%-50.0%]). Over a lifetime, anatomic approaches gained an additional 6 months in perfect health compared with functional testing (CTA, 25.16 [95% CI, 25.14-25.19] QALYs; CTA with FFR_CT_, 25.14 [95% CI, 25.12-25.17] QALYs; functional testing, 24.68 [95% CI, 24.66-24.70] QALYs). Anatomic strategies were less costly and more effective; thus, CTA with FFR_CT_ dominated and CTA alone was cost-effective (ICERs ranged from $1912/QALY for women and $3,559/QALY for men) compared with functional testing. In probabilistic sensitivity analyses, anatomic approaches were cost-effective in more than 65% of scenarios, assuming a willingness-to-pay threshold of $100 000/QALY.

**Conclusions and Relevance:**

The results of this study suggest that anatomic strategies may present a more favorable initial diagnostic option in the evaluation of low-risk SCP compared with functional testing.

## Key Points

**Question:**

Are first-line anatomic approaches to low-risk stable chest pain evaluation cost-effective compared with functional testing?

**Findings:**

In this cost-effectiveness analysis using an individual-based Markov microsimulation model based on 10 003 participants in a randomized clinical trial, anatomic approaches were cost-effective compared with functional testing across a wide range of variations in clinical care and patient characteristics. Adding fractional flow reserve to coronary computed tomography angiography resulted in modest improvements after the initially increased costs of care were offset by fewer and more targeted coronary revascularizations.

**Meaning:**

These findings suggest that anatomic strategies may present a favorable initial diagnostic option in the evaluation of low-risk stable chest pain compared with functional testing.

## Teaser

This economic evaluation determines whether anatomic approaches are cost-effective compared with functional tests for the assessment of low-risk stable chest pain.

## Introduction

Annually, more than 8.7 million patients undergo noninvasive diagnostic testing for suspected coronary artery disease (CAD) at an expense of $15 billion in the United States.^1^ Nearly all of these tests target functional assessment of myocardial ischemia (64%, nuclear imaging with single photon emission computed tomography [SPECT]; 31%, stress echocardiography).^2,3^ However, the positive predictive value of these tests for anatomically obstructive CAD in patients referred to invasive coronary angiography (ICA) remains low (38%).^4^ Meanwhile, coronary computed tomography angiography (CTA), a test permitting noninvasive visualization of CAD, is currently performed in less than 5% of chest pain evaluations.

Randomized comparisons between functional and anatomic index testing in low-risk stable chest pain (SCP) (ie, the Prospective Multicenter Imaging Study for Evaluation of Chest Pain [PROMISE]^5^ and the Scottish Computed Tomography of the Heart [SCOT-HEART]^6^ trials), have had mixed results. The PROMISE trial^5^ reported no differences between anatomic and functional evaluation strategies in SCP for incident major adverse cardiovascular events (MACE) after 2 years, while the SCOT-HEART study^6^ showed a 41% reduction in nonfatal myocardial infarction (MI) for patients randomized to coronary CTA compared with functional testing after 5 years. In addition, both trials reported higher referral rates to invasive coronary angiography (ICA) and subsequent revascularization after 2 years, with SCOT-HEART reporting similar ICA and revascularizations rates between the 2 strategies after 5 years.^5,7^ Based on these data, the 2019 European Guidelines for the evaluation of patients with SCP^8^ increased the level of recommendation for coronary CTA to I class B. Recent data support adding fractional flow reserve based on standard resting coronary CTA (FFR_CT_) in patients with intermediate stenosis (ie, 30%-69%), given that it leads to a 2-fold increase in specificity over anatomic assessment with coronary CTA alone (74% vs 34%) compared with the criterion standard, invasive FFR.^9^ To clarify the potential long-term health and economic implications of initial anatomic and functional diagnostic approaches to patients with low-risk SCP, we developed a Markov microsimulation model based on individual patient-level data from the PROMISE trial.

## Methods

### Model Overview

**Figure 1.**
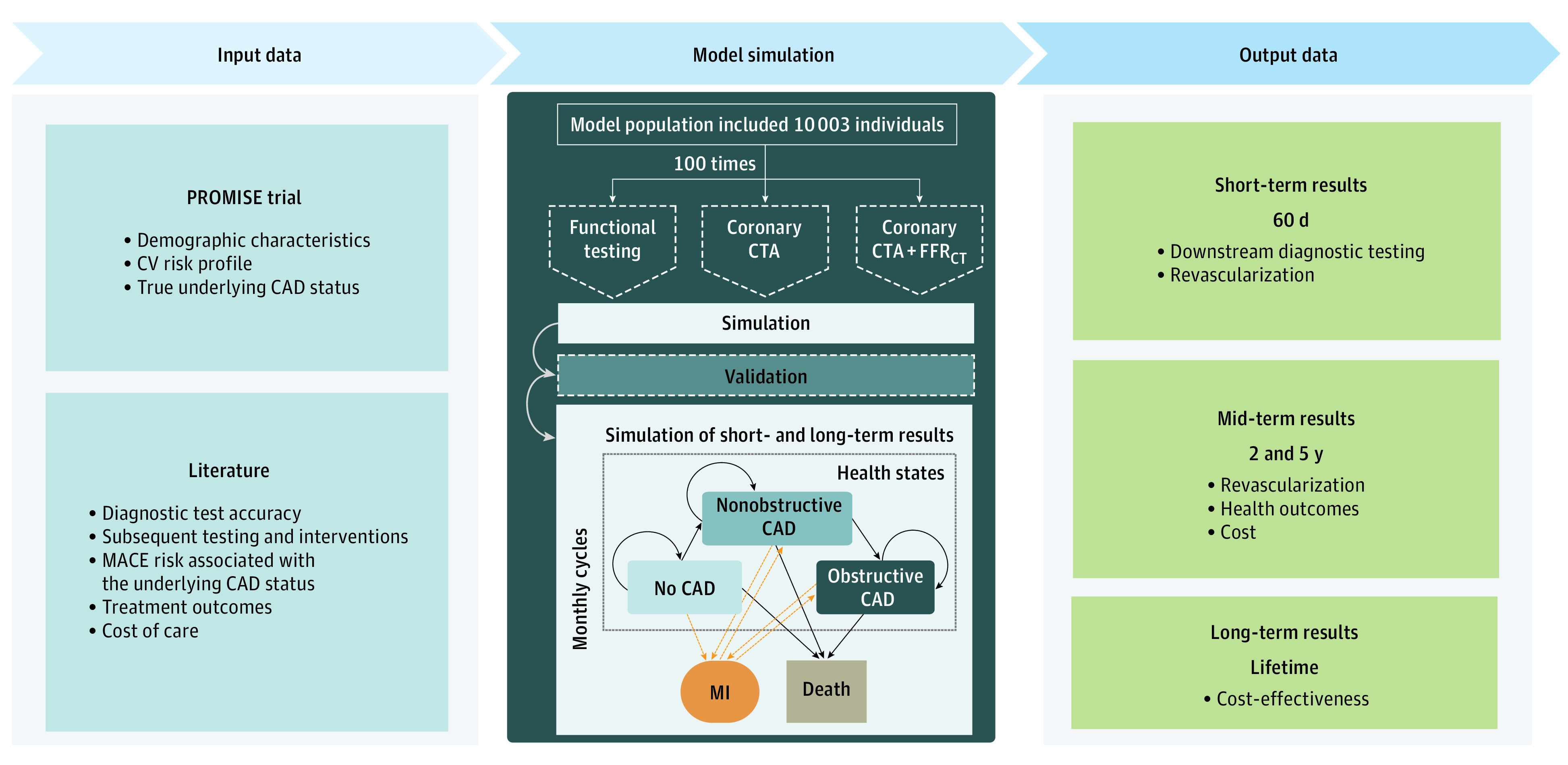
Individual-Based Markov Microsimulation Model Overview and Lifetime Outcomes Baseline population characteristics, risk factors, and underlying true coronary artery disease (CAD) status was observed in the Prospective Multicenter Imaging Study for Evaluation of Chest Pain (PROMISE) study,^5^ whereas diagnostic test accuracy, baseline rules for further testing and interventions, major adverse cardiovascular event (MACE) risk associated with the underlying CAD status, treatment effects, and cost of care were taken from the literature. After simulation of the 60-day and 2-year functional testing and coronary computed tomography angiography (CTA) results, model accuracy was validated by comparing model simulated with observed patient management, health outcomes, and costs. Next, simulation of short-term and long-term outcomes of the model population after undergoing the index tests (coronary CTA, functional testing, or CTA with fractional flow reserve based on standard resting CTA [FFR_CT_]) by modeling health states (no CAD, nonobstructive CAD, or obstructive CAD) and transitions within in monthly cycles until end of life. Model outcomes were downstream diagnostic testing and revascularization rate in the short term; revascularization, health outcomes, and cost during 2 and 5 years; and cost-effectiveness over lifetime. CV indicates cardiovascular; MI, myocardial infarction.

We developed a Markov microsimulation model using individual patient data from the PROMISE trial^5^ for the following 3 strategies: coronary CTA, coronary CTA with FFR_CT_, and functional testing. Each patient entered the model 100 times with a health state defined by their underlying CAD status (ie, no CAD, nonobstructive CAD, or obstructive CAD) and underwent different life cycles and disease progression based on probabilities. The likelihood of positive test results, referral to ICA and subsequent revascularization, statin therapy, and related benefits that translated into different risk of MACE were simulated based on the initial correct diagnosis of CAD and CAD progression. The model was validated by comparing model outcomes with outcomes observed in PROMISE. The validated model was used to simulate short-term, mid-term, and long-term health and economic outcomes and cost-effectiveness over a lifetime (Figure 1). The PROMISE trial was accepted by local or central institutional review boards, and all participants provided written informed consent. We applied good modeling practices as suggested by the ISPOR-SMDM modeling task force,^10^ including calibration to observed data, using approaches developed in prior work and following consensus guidelines, such as the Consolidated Health Economic Evaluation Reporting Standards (CHEERS) reporting guideline.^11,12,13,14,15^

This cost-effective analysis is based on individual patient-level demographic characteristics and risk factors from 10 003 real-life US patients from 192 US sites presenting with suspicion of obstructive CAD. This population was represented 100 times in the model baseline population, allowing us to model the course of life for each participant with 100 variations, considering many different scenarios based on the probability for a medical action or an event to occur.

### Model Input Parameters

#### Patient Demographic Characteristics, Cardiovascular Risk Profile, Index Testing, and CAD Status

Baseline patient demographic characteristics, cardiovascular (CV) risk profiles, and CAD status were taken from patient-level data of the 10 003 patients enrolled in the PROMISE trial.^5^ The true underlying CAD status was determined by using expert core laboratory test readings as the criterion standard. The CAD finding of each index test at baseline was derived based on the diagnostic accuracy, as recommended by the European Society of Cardiology (ESC) Guidelines (eTable 1 in the Supplement).^16^ Because PROMISE was a randomized trial, input of distribution of presence and extent of CAD was similar for patients randomized to anatomic and functional testing groups (eAppendix in the Supplement).

#### Downstream Testing

ICA was indicated in 3 cases. They were (1) large territory of reversible myocardial ischemia by functional testing; (2) 70% luminal stenosis in at least 1 vessel or 50% luminal narrowing in the left main (LM) coronary artery by coronary CTA, and (3) a hemodynamically significant stenosis with an FFR_CT_ of 0.8 or less in patients with at least 1 luminal stenosis of 30% to 69% (eTable 2 in the Supplement).^9,16,17^

#### Medical Treatment

Medical treatment, with the exception of statin therapy, was similar for all strategies and defined by the American Heart Association/American College of Cardiology (AHA/ACC) Guidelines for the management of SCP^18^ and thus did not lead to any differences in health outcomes (eTable 3 in the Supplement). We focused on simulating potential differences in outcomes among the 3 index tests to identify the presence and extent of underlying CAD. Patients with a diagnosis of obstructive or nonobstructive CAD (limited to anatomic strategies) were statin eligible. Statin therapy was further indicated for patients with at least a 7.5% atherosclerotic CV disease (ASCVD) risk score, per SCP guidelines. Based on the JUPITER trial,^19^ the model assumed that lifelong statin therapy was associated with a 65% risk reduction for MI and 20% risk reduction for CV mortality. For all tests, a missed diagnosis of CAD resulted in loss of benefits of statin therapy. The treatment effect was modeled to reflect differences in hazard ratios between no CAD, nonobstructive CAD, and obstructive CAD.^17^

#### Coronary Revascularization

Based on the 2014 ACC/AHA Guidelines on the treatment of patients with stable ischemic heart disease, patients with significant LM stenosis (>50%) and those with 3-vessel disease in ICA underwent coronary artery bypass grafting (CABG), whereas patients with 1- or 2-vessel disease underwent percutaneous coronary intervention (PCI).^18^ The treatment effect was considered similar for optimal medical therapy and coronary revascularization based on the COURAGE trial.^20^

#### Health States, CAD Progression, and Health Outcomes

Each patient entered the model with a health state defined by their underlying CAD status (ie, no CAD, nonobstructive CAD, obstructive CAD). Progression of CAD was modeled as a function of baseline CAD status, age, sex, and National Cholesterol Education Program risk score from a cohort of patients with SCP using a simulated annealing approach (eAppendix and eFigure 1 in the Supplement).^21,22^ Patients were simulated to either remain in the same health status (no change in CAD) or to transition from 1 health state to another over time depending on the past (progression of CAD) in monthly cycles until the end of life. Findings of the index diagnostic evaluation (dependent on the diagnostic accuracy of each test) and CV risk profile determined downstream testing, statin therapy, and related benefits. The likelihood of experiencing MACE in each monthly cycle with a given CAD status was modeled based on the CONFIRM registry, and the risk of all-cause death was derived from US life tables (eTable 4 in the Supplement).^23,24,25,26,27,28,29,30^ The risk of periprocedural mortality during diagnostic ICA, PCI, and CABG was simulated for each invasive procedure.^31,32,33,34,35,36,37^

#### Costs of Care

Cost of diagnostic tests (coronary CTA, $404; functional testing, $174-$1061; ICA, $3656) and interventions (PCI, $12 779; CABG, $32 546) are expressed in 2014 US dollars and were taken from the PROMISE trial.^15^ The cost of FFR_CT_ was $1450, per current US Centers for Medicare & Medicaid Services website.^38^ Cost of medications was based on the 2017 edition of the Red Book (eTable 5 in the Supplement).^39^

### Study End Points

This study had 4 end points. They were (1) rates of diagnostic ICA and revascularization-to-ICA ratio at 60 days; (2) rate of coronary revascularization (PCI or CABG) at 60 days, 2 years, 5 years, and over lifetime; (3) MACE (MI, CV mortality), all-cause mortality, and the composite endpoint at 2 years, 5 years, and lifetime; and (4) cost-effectiveness, defined as cost and quality-adjusted life-years (QALYs) at 2 years, 5 years, and over a lifetime, and incremental cost-effectiveness ratio (ICER) and life-years gained over lifetime (eAppendix in the Supplement). ICERs were calculated in accordance with cost-effectiveness analysis guidelines and were expressed as cost per QALY. A strategy was considered cost-effective when the ICER was less than $100 000/QALY.^40^ A strategy that was both less costly and more effective than another was defined as *dominant*.^11,12,13,14,41^ ICER values were based on costs and QALYs that were each discounted at 3% per year, as recommended by the US Panel on Cost-effectiveness in Health and Medicine.^42,43^

### Model Validation: Coronary CTA and Functional Testing in PROMISE

The model was validated by comparing model outcomes with real-life events reported in PROMISE, including test results, referral to ICA, coronary revascularization, incident MACE, and costs during 60 days and 2 years. The purpose of the validation was to ensure that the model was well calibrated and stable, thereby ensuring confidence for simulations beyond the 2-year follow-up period of PROMISE (Figure 1).

### Subgroup and Sensitivity Analyses

We conducted 2 subgroup analysis; to assess the robustness of ICER analyses, we tested cost-effectiveness outcomes across subgroups, stratified by (1) sex and (2) being younger or older than the median (ie, 60 years).^44,45^ We also conducted 4 sensitivity analyses: (1) adherence to medical therapy, a scenario of 5 years of full adherence followed by 5 years of declining adherence (in monthly steps with no patients receiving statins after 10 years) and another scenario with full adherence for 5 years and no medical treatment effect afterwards; (2) to assess whether adding functional information to anatomical stenosis would substantially affect the rate of invasive testing among those with luminal narrowing greater than 70%, we expanded the indication of FFR_ct_ to include such patients; (3) do nothing strategy, in which patients only received medication according to their risk factor profile^46^; and (4) to visualize the heterogeneity and thus the uncertainty created by our 1 000 300 microsimulation cases per strategy, we conducted a quasi–probabilistic sensitivity analysis (PSA) and calculated cost-effectiveness acceptability curves for CTA alone and CTA with FFR_CT_ compared with functional testing. Cost and QALY distributions for the quasi-PSA were informed by parameter estimates from our data. Results from the quasi-PSA were then used to calculate cost-effectiveness acceptability curves.

### Statistical Analysis

The model was analyzed from the societal perspective of the United States. For each strategy, we simulated each PROMISE participant 100 times (ie, each of the 10 003 PROMISE patients entered the model 100 times for each strategy, resulting in 1 000 300 observations per strategy). This enabled us to generate standard errors for the cost and effectiveness end points that were small enough to generate stable estimates of the effect sizes of interest, ensuring that the difference in QALYs and costs between the interventions was at least 2 times greater than the standard error of the difference. Thus, all comparisons are reported without *P* values. The model was programmed in TreeAge Pro Suite (TreeAge Software). All data and statistical analyses were performed using Stata version 14.2 (StataCorp).

## Results

### Patient Population

The model cohort had identical individual patient demographic characteristics, including age, sex, race, and CV risk factors, as the 10 003 individual patients who participated in the PROMISE trial^5^ (Table 1). The median (interquartile range) age was 60.0 (54.4-65.9) years, 5270 (52.7%) were women, and 7693 (77.7%) were White individuals. The population had a substantial CV risk factor burden: 2531 (25.3%) had a CAD risk equivalent, and 6697 (67.6%) had a 10-year risk of events of at least 7.5%. The mean (SD) pretest likelihood of obstructive CAD according to a combined Diamond and Forrester and Coronary Artery Surgery Study model was 53.3% (21.4).

**Table 1.** Demographic Characteristics and CV Risk and 2-Year MACE in Patients With Stable Chest Pain in the Markov Model^a^

Variable	No. (%)
Age, median (IQR), y	60.0 (54.4-65.9)
Women	5270 (52.7)
Race	
White	7693 (77.7)
Black	1071 (10.8)
Other	1239 (12.4)
CV risk factors	
Body mass index, mean (SD)^b^	30.5 (6.1)
Hypertension	6501 (65.0)
Diabetes	2144 (21.4)
Dyslipidemia	6767 (67.7)
Family history of premature CAD	3202 (32.1)
PAD or cerebrovascular disease	552 (5.5)
CAD risk equivalent	2531 (25.3)
Metabolic syndrome	3772 (37.7)
Current or past tobacco use	5104 (51.0)
Sedentary lifestyle	4866 (48.8)
History of depression	2058 (20.6)
Risk burden	
No risk factors	263 (2.6)
Risk factors per patient, mean (SD), No.	2.4 (1.1)
Combined Diamond and Forrester and Coronary Artery Surgery Study Risk score, mean (SD), %	53.3 (21.4)
Framingham risk score categories	
Low risk, <6%	686 (6.9)
Intermediate risk, 6%-20%	5114 (51.2)
High risk, >20%	4188 (41.9)
Framingham risk score, median (IQR)	17.1 (10.6-28.6)
ASCVD risk	
Low risk, <7.5%	3204 (32.4)
Elevated risk, ≥7.5%	6697 (67.6)
Median (IQR)	11.3 (6.1-19.8)
Chest pain type	
Angina	
Typical	1166 (11.7)
Atypical	7773 (77.7)
Nonanginal pain	1064 (10.6)
MACE during a median follow-up of 25 mo	
CV death or MI	157 (1.6)
MI	70 (0.7)
CV death	35 (0.4)
Death from any cause	149 (1.5)
Death or MI	216 (2.2)

Abbreviations: ASCVD, atherosclerotic cardiovascular disease; CAD, coronary artery disease; CV, cardiovascular; IQR, interquartile range; MACE, major adverse cardiovascular event; MI, myocardial infarction; PAD, peripheral artery disease.

^a^ Patient characteristics of the 1 000 300 modeled individuals were simulated based on individual patient data from the Prospective Multicenter Imaging Study for Evaluation of Chest Pain trial^5^; therefore, they are identical to the original PROMISE cohort.

^b^ Body mass index was calculated as weight in kilograms divided by height in meters squared.

### Model Validation

**Figure 2.**
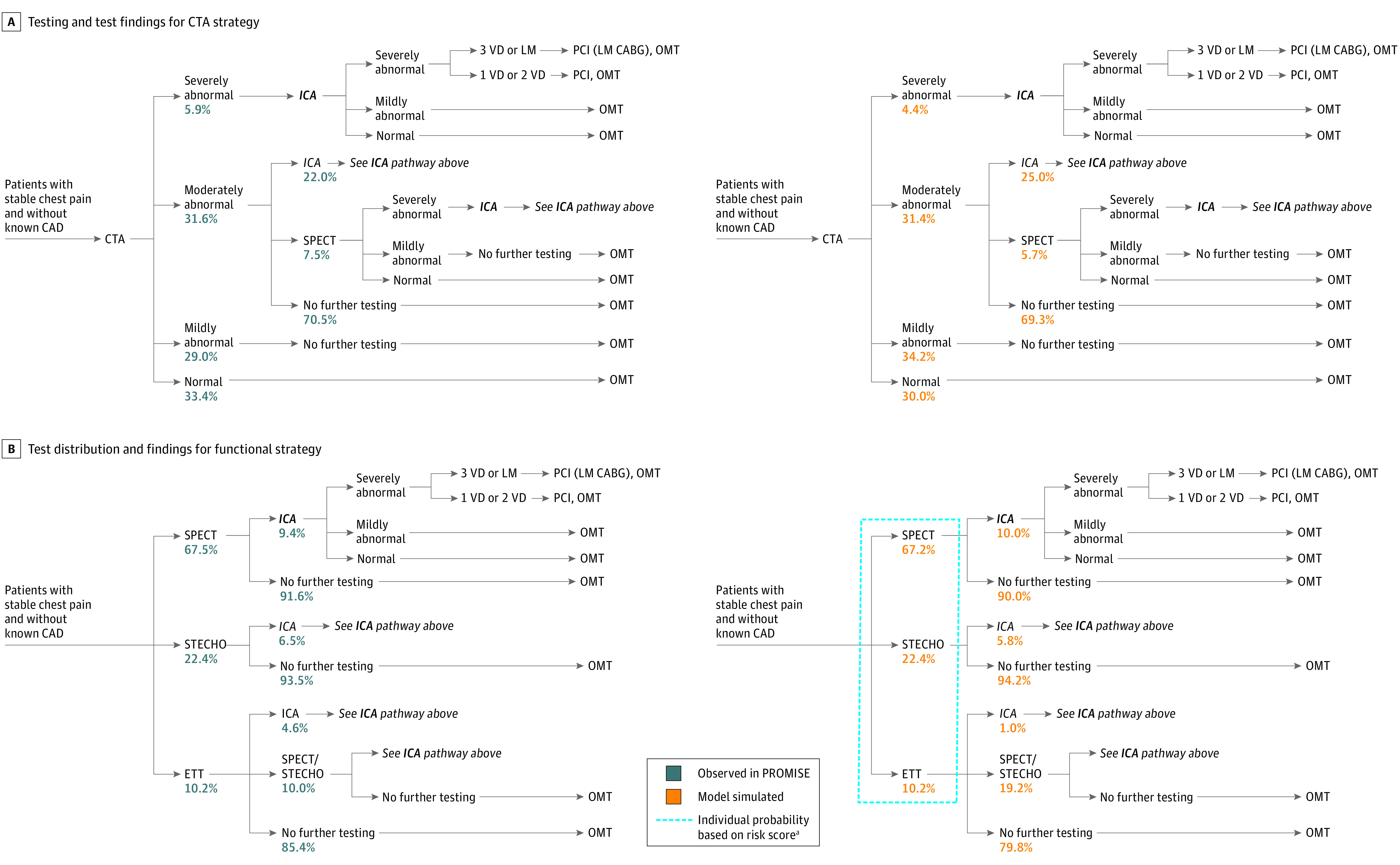
Comparison of Observed vs Simulated Rate of Testing and Test Findings for Coronary Computed Tomography Angiography (CTA) Strategy and Test Distribution and Findings for Functional Strategy A,Data based on site and core laboratory test readings. B, Average pathway probabilities as observed in PROMISE vs as model simulated, in which patients underwent pathways according to their risk score, ie, if patient is at lower risk, then there is a higher probability that the given patient will be tested with exercise treadmill test (ETT). An invasive coronary angiography (ICA) finding of severely abnormal indicated coronary artery disease (CAD) with at least 70% stenosis; mildly abnormal, nonobstructive CAD with 1% to 70% stenosis; normal, no stenosis. CTA, single photon emission computed tomography (SPECT), stress echocardiography findings (STECHO), and ETT are defined in eTable 2 in the Supplement.^17^ CABG indicates coronary artery bypass grafting; LM, left main disease; OMT, optimal medical treatment; PCI, percutaneous coronary intervention; PROMISE, Prospective Multicenter Imaging Study for Evaluation of Chest Pain; and VD, vessel disease.

First, we modeled the assignment of the different functional testing alternatives used in PROMISE, resulting in accurate estimations for stress SPECT (67.5% [95% CI, 66.2%-68.8%] vs 67.2% [95% CI, 67.1%-67.3%]), stress echocardiography (22.4% [95% CI, 21.2%-23.7%] vs 22.5% [95% CI, 22.5%-22.6%]), and exercise treadmill testing (10.2% [95% CI, 8.9%-11.5%] vs 10.4% [95% CI, 10.3%-10.5%]) for modeled vs observed PROMISE data, respectively. Similarly, the model, compared with PROMISE data, accurately simulated test results (eg, coronary CTA with 30%-69% stenosis: 31.6% [95% CI, 30.3%-32.9%] vs 31.4% [95% CI, 31.3%-31.5%]; functional testing with inducible myocardial ischemia: 8.8% [95% CI, 8.0%-9.6%] vs 7.9% [95% CI, 7.8%-8.0%]) (Figure 2A and Figure 2B) and ICA and coronary revascularization rates (coronary CTA: ICA, 12.2% [95% CI, 10.9%-13.5%] vs 12.3% [95% CI, 12.2-12.4%]; revascularization, 6.2% [95% CI, 5.5%-6.9%] vs 6.4% [95% CI, 6.3%-6.5%]; functional strategy: ICA, 8.1% [95% CI, 7.4%-8.9%] vs 8.2% [95% CI, 8.1%-8.3%]; revascularization, 3.2% [95% CI, 2.7%-3.7%] vs 3.3% [95% CI, 3.2%-3.4%]). Lastly, the model accurately predicted costs compared with observed costs (coronary CTA, $2494 vs $2546; functional strategy, $2240 vs $2189) and 2-year MACE rates (coronary CTA, 2.1% [95% CI, 1.7%-2.5%] vs 2.3% [95% CI, 2.2%-2.4%]; functional strategy, 2.2% [95% CI, 1.8%-2.6%] vs 2.4% [95% CI, 2.3-2.4%]) (eTable 6 in the Supplement).

### Comparison of Coronary CTA, CTA With FFR_CT_, and Functional Testing Strategies

#### Short-term Outcomes

Overall, 3141 patients (31.4%) had a 30% to 69% stenosis on coronary CTA and underwent CTA with FFR_CT_. Based on ASCVD risk score and diagnostic test results, 6702 patients (67.0%) per functional strategy, 8539 (85.4%) per coronary CTA, and 8552 (85.5%) per CTA with FFR_CT_ were eligible for statin treatment. Because of the higher sensitivity of coronary CTA to detect CAD, the frequency of ICA and coronary revascularization was higher for patients who underwent coronary CTA and CTA with FFR_CT_ compared with those who underwent functional testing (ICA: 12.3% [95% CI, 12.3%-12.4%] and 10.5% [95% CI, 10.5%-10.6%] vs 8.1% [95% CI, 8.0%-8.1%]; revascularization: 6.6% [95% CI, 6.6%-6.7%] and 6.3% [95% CI, 6.3%-6.4%] vs 3.3% [95% CI, 3.3%-3.4%]) (eFigure 2 in the Supplement). The revascularization-to-ICA ratios for CTA with FFR_ct_ and CTA approaches were higher compared with functional testing, indicating a more effective patient selection for ICA (59.5% [95% CI, 59.2%-598.8%] and 53.7% [95% CI, 53.3%-54.0%] vs 40.7% [95% CI, 40.4%-50.0%]) (eFigure 2 in the Supplement).

#### Mid-term Outcomes

The 2-year revascularization rates for coronary CTA alone and CTA with FFR_CT_ were nearly twice as high as those for functional testing (6.6% [95% CI, 6.5%-6.6%] and 6.3% [95% CI, 6.3%-6.4%] vs 3.6% [95% CI, 3.6%-3.7%]) and remained higher after 5 years, although the functional strategy saw the highest relative increase (functional testing, 21.0% [95% CI, 20.9%-21.1%]; coronary CTA, 2.9% [95% CI, 2.8%-3.0%]; coronary CTA with FFR_CT_, 3.1% [95% CI, 3.0%-3.2%]) (Table 2). The MACE rate in this low-risk SCP population was low across all strategies, not exceeding 1.5% after 2 years and 3.9% after 5 years. Higher costs of anatomic approaches after 2 and 5 years were mainly associated with the higher ICA and revascularization rates. The additional cost of FFR_CT_ ($1450) was offset by fewer ICAs and revascularizations after 5 years compared with coronary CTA alone. Anatomic approaches had higher QALYs at both 2 and 5 years: the QALY gains for CTA with FFR_CT_ and for CTA alone were 0.12 (*P* < .001) and 0.13 (*P* < .001), respectively, or 1.5 months of longer life in perfect health (Table 2).

**Table 2.** Model-Derived Coronary Revascularization and MACE Rates at 2 and 5 Years and Over Lifetime, by Index Test Strategy

Index test	2-y Rate			5-y Rate			Lifetime rate		
	Coronary CTA	CTA with FFR_CT_^a^	Functional testing	Coronary CTA	CTA with FFR_CT_^a^	Functional testing	Coronary CTA	CTA with FFR_CT_^a^	Functional testing
Revascularization, %									
Any revascularization	6.59	6.33	3.62	6.78	6.53	4.38	12.59	12.44	13.33
PCI	4.59	4.33	2.95	4.71	4.45	3.59	8.41	8.24	10.40
CABG	2.00	2.00	0.67	2.07	2.08	0.79	4.18	4.20	2.93
MACE, %									
CV death or MI	1.11	1.10	1.44	3.05	3.05	3.89	48.61	48.82	51.83
MI	0.75	0.73	0.90	1.92	1.92	2.29	14.34	14.41	15.11
CV death	0.37	0.39	0.55	1.19	1.20	1.68	42.13	42.30	44.89
Death from any cause	1.42	1.42	1.61	4.12	4.11	4.61	100.00	100.00	100.00
Death or MI	2.16	2.13	2.49	5.95	5.93	6.78	100.00	100.00	100.00
Cost/patient (95% CI), $	2808 (2796-2821)	2998 (2985-3010)	2404 (2396-2413)	3276 (3262-3290)	3251 (3237-3265)	2759 (2749-2770)	8683 (8652-8713)	7222 (7192-7252)	7989 (7958-8020)
QALY/patient (95% CI)	1.869 (1.869-1.870)	1.870 (1.869-1.870)	1.867 (1.867-1.867)	4.610 (4.609-4.611)	4.611 (4.610-4.612)	4.598 (4.597-4.599)	25.162 (25.139-25.185)	25.143 (25.120-25.166)	24.680 (24.657-24.704)

Abbreviations: CABG, coronary artery bypass grafting; CTA, computed tomography angiography; CV, cardiovascular; FFR_CT_, noninvasive fractional flow reserve derived from computed tomography; MACE, major adverse cardiovascular event; MI, myocardial infarction; PCI, percutaneous coronary intervention; QALY, quality-adjusted life-years.

^a^ FFR_CT_ performed in patients with 30% to 69% stenosis as detected by coronary CTA.

#### Long-term Outcomes

There was a significant dynamic in coronary revascularizations, costs, and QALYs between mid-term and lifetime follow-up. Over a lifetime, the model estimated similar frequency of coronary revascularizations across all strategies (Table 2). As a result, differences in costs between the anatomic and functional approaches decreased. Over a lifetime, anatomic approaches had significantly higher QALYs compared with functional testing (QALY gain for CTA with FFR_CT_: 0.46; CTA alone: 0.48; indicating 6 months of longer life in perfect health). Over a lifetime, the coronary CTA strategy alone was cost-effective compared with functional testing (ICER: $2743/QALY), and the CTA with FFR_CT_ strategy was less costly and more effective and thus dominated functional testing (Table 3). Modeling different accuracies for CTA and FFR_CT_ by assuming worse performance due to the outdated CT technology used in the PROMISE trial did not alter the results of the main analysis.

**Table 3.** Cost, QALYs, ICER, and Life-Years Gained From Coronary CTA and Coronary CTA With FFR_CT_ Compared With Functional Testing

Strategy	Cost (95% CI), $		QALY (95% CI)		Discounted ICER ($/QALY)^b^	Life-years gained (95% CI), y
	Undiscounted	Difference^a^	Undiscounted	Difference^a^		
**Coronary CTA vs functional testing**						
Functional strategy	7989 (7958 to 8020)	NA	24.68 (24.66 to 24.70)	NA	NA	26.51 (26.48 to 26.53)
Coronary CTA strategy	8683 (8652 to 8713)	694 (660 to 728)	25.16 (25.14 to 25.19)	0.48 (0.46 to 0.50)	2743^c^	27.03 (27.00 to 27.05)
**Coronary CTA with FFR_CT_ vs functional testing**						
Functional strategy	7989 (7958 to 8020)	NA	24.68 (24.66 to 24.70)	NA	Dominated^d^	26.51 (26.48 to 26.53)
CTA with FFR_CT_ strategy	7222 (7192 to 7252)	−767 (−805 to −729)	25.14 (25.12 to 25.17)	0.46 (0.44 to 0.49)	NA	27.01 (26.99 to 27.04)

Abbreviations: CTA, computed tomography angiography; FFR_CT_, noninvasive fractional flow reserve derived from computed tomography; ICER, incremental cost-effectiveness ratio; NA, not applicable; QALY, quality-adjusted life-years.

^a^ Cost and QALY differences are expressed in reference to functional strategy.

^b^ Discounted at 3% annually, as recommended by the US Panel on Cost-Effectiveness in Health and Medicine.^42,43^

^c^ A strategy is considered cost-effective when the ICER is less than $100 000/QALY.^40^

^d^ A strategy is considered dominated by the other if the other has lower cost and higher QALY.

### Sensitivity Analyses

#### Subgroup Analyses

Compared with functional strategy, coronary CTA remained cost-effective with an ICER in women and men as well as in individuals older than and younger than the median age of 60 years (ICER range, $1912/QALY for women to $3559/QALY for men). CTA with FFR_CT_ was cost-effective in men (ICER, $192/QALY) but dominated the functional strategy across other subgroups (eTable 7 in the Supplement).

#### Adherence to Medical Therapy

Modeling a continuous decline in statin therapy adherence after 5 years, the lifetime cost of coronary CTA strategy decreased to $6438 (95% CI, $6413-$6464) but also resulted in the loss of health benefits and thus yielded lower QALY (QALY difference, 0.12; 95% CI, 0.10-0.14). However, coronary CTA remained cost-effective compared with functional strategy (ICER, $2927/QALY). Similar results were seen for a CTA with FFR_CT_ strategy. Modeling complete nonadherence to statin therapy for anatomical strategies after 5 years resulted in the loss of some of the observed health benefits compared with functional testing but still lower MACE rates for anatomic strategies compared with functional testing (CTA alone and CTA with FFR_CT_ vs functional testing, MACE rate: 52.5% [95% CI, 52.4%-52.6%] and 52.7% [95% CI, 52.6%-52.8%] vs 53.3% [95% CI, 53.2%-53.4%]). However, anatomic approaches were still cost-effective compared with functional testing (CTA alone, $2291/QALY; CTA with FFR_CT_, $2723/QALY), mostly because of the decreased costs of care.

#### Expanding the Indication of FFR_CT_ to Patients With Greater Than 70% Luminal Narrowing

Expanding the use of FFR_CT_ to the 4.4% of patients who had greater than 70% stenosis resulted in a downward reclassification and avoidance of ICA in 17.8% (95% CI, 16.6%-19.0%) of these patients. At 60 days, this would lead to an overall decrease of ICA by 0.8% (from 10.5% [95% CI, 10.3%-10.7%] to 9.7% [95% CI, 9.5%-9.9%]) and coronary revascularizations (from 6.3% [95% CI, 6.1%-6.5%] to 5.5% [95% CI, 5.4%-5.6%]) in the overall population and a 4.4% increase of the size of the FFR_CT_ group. Over a lifetime, results are very similar compared with the main analysis, resulting in lower cost and higher QALYs for coronary CTA and FFR_CT_ strategy compared with the functional testing strategy.

#### Do Nothing Strategy

A do nothing strategy resulted in the lowest cost and lowest QALYs compared with all other strategies; all strategies were cost-effective compared with do nothing, assuming a cost-effectiveness threshold of $100 000/QALY. However, functional testing was only slightly below the threshold (functional strategy vs do nothing, $99 678/QALY; CTA with FFR_CT_ vs do nothing, $36 968/QALY; CTA vs do nothing, $59 436/QALY).

#### Quasi-PSA

When each outcome was expressed in incremental costs and incremental effects, anatomic approaches remained less costly and more effective compared with functional strategy in 38.6% of scenarios for coronary CTA and in 51.5% of scenarios for CTA with FFR_CT_ (eFigure 3A and eFigure 3B in the Supplement). Assuming the willingness to pay is $100 000/QALY, the probability that the coronary CTA strategy and CTA with FFR_CT_ remained cost-effective compared with functional testing was 69.4%, and 65.4%, respectively (eFigure 3C and eFigure 3D in the Supplement).

## Discussion

There is heterogenous data on the appropriate choice of diagnostic index testing in the evaluation of low-risk SCP.^5,6^ The results of our analysis, using a Markov model incorporating individual patient-level data from PROMISE, suggest that anatomic approaches are cost-effective compared with functional testing across a wide range of assumptions in clinical care and patient characteristics, mostly because of a higher sensitivity to detect nonobstructive and obstructive CAD and the ability to tailor statin therapy accordingly. Adding FFR_CT_ to coronary CTA resulted in further, although modest, improvements, and the initial higher costs were offset by fewer and more targeted coronary revascularizations. In PSAs, anatomic approaches were cost-effective in most scenarios assuming a willingness-to-pay threshold of $100 000/QALY. Overall, our results support the new ESC guidelines, suggesting that anatomic strategies may present a favorable initial diagnostic option in the evaluation of low-risk SCP compared with functional testing.

This analysis sought to illuminate the effects of differences in the diagnostic capability to detect nonobstructive and obstructive CAD between functional and anatomic approaches on identifying patients who are statin eligible and those eligible for referral to coronary revascularization. In addition, our model included FFR_CT_ as an emerging testing option. Our model, similar to PROMISE and SCOT-HEART, showed overall low rates of ICA and coronary revascularization within 2 and 5 years for all strategies but with higher rates for anatomic approaches compared with functional testing (12.3% and 10.5% vs 8.1% for ICA, respectively, and 6.6% and 6.3% vs 3.3% for revascularization, respectively). This observation, in line with widely published data,^39,47,48,49^ appeared to be driven by the higher sensitivity of anatomic testing to detect CAD. Furthermore, optimized patient selection for ICA and subsequent coronary revascularization was shown for FFR_CT_, which reclassified intermediate lesions with a luminal narrowing of 30% to 69%^9,16,17^ (revascularization-to-ICA ratio: CTA with FFR_CT_, 59.5%; CTA strategy, 53.7%; functional testing, 40.7%), consistent with previous observational studies (revascularization-to-ICA ratio for FFR_CT_ in the ADVANCE registry,^50^ 59.5%; PLATFORM study,^51^ 58.3%). However, a relatively small change was observed for CTA with FFR_CT_ strategy, resulting in a 14.6% reduction of the ICA rate. This observation is may be surprising but can be explained by the fact that only 31% of patients received FFR_CT_, and the positivity rate was very low—similar to absolute rates of revascularization in this population. Interestingly, the additional cost of FFR_CT_ (ie, $1450) was offset after 5 years by fewer ICAs and revascularizations compared with coronary CTA alone. Expanding the indication of FFR_CT_ for those with luminal narrowing of greater than 70% affected very few patients (0.8%). Hence, although a sizeable portion of those with stenosis (17.8% of these patients) was reclassified and downgraded by FFR_CT_^52^ and those patients could avoid ICA and unnecessary coronary revascularization, this affected only 0.8% of all patients. Understandably, this change of management did not alter the results of the cost-effectiveness analysis in the overall population significantly.

Our second focus was to determine how tailoring statin therapy to the presence and extent of CAD would affect cost-effectiveness. Assuming similar optimal medical treatment, except for statin therapy, for all strategies constitutes an important simplification of the model but was justified because differences in test findings mainly affected statin therapy. Close to the 41% reduction in MACE observed in SCOT-HEART, our model estimated that MACE at 2 and 5 years was 23.6% and 21.6% higher after functional testing compared with anatomic strategies after 2 and 5 years, respectively. This was associated with the difference in diagnosis of nonobstructive and obstructive CAD (for which anatomic strategies have better diagnostic accuracy^16,17,53,54^) and consequent differences in statin treatment (67% for functional testing vs 85% for anatomic approaches). Our estimates of the differences of statin effects are possibly conservative because we assumed full adherence of all patients in the functional arm, putting two-thirds of that population already on statin treatment (compared with 57% in SCOT HEART^6^ and 50% in PROMISE).^55^ In this context, it is important to compare our assumptions and results with published data. Notably, unlike any other prior cost-effectiveness analyses of statin therapy, we were able to tailor the benefits of statins, ie, an overall reduction in mortality by 20%, to the underlying CAD, assuming 0% mortality reduction for those without CAD, 30% for those with nonobstructive CAD, and 30% for obstructive CAD. Reassuringly, this is in line with reports from several statin trials, including the JUPITER and 4S studies (30% mortality reduction in patients with coronary heart disease).^56,57^ Moreover, our reported gain of 0.5 additional QALY for anatomical strategies vs functional testing appears to be comparable with the 0.28 additional QALY reported in the 4S cost-effectiveness analysis, once we consider that patients were assumed to receive treatment for only 5 years in 4S and after that no statin effect was modeled. Compared with the JUPITER cost-effectiveness analysis, our discounted (3% per year) lifetime QALY difference was 0.24 (instead of the approximately 0.5 when using undiscounted values) and thus less than the 0.31 reported in JUPITER, in which they compared potent statin therapy with placebo.^53^ Moreover, the JUPITER cost-effectiveness analysis assumed 15 years of treatment, while we assumed statin treatment over a lifetime. In their sensitivity analyses, a maximum treatment duration of 25 years led to an ICER reduction of 20% (from $25 000 to $20 000). Because the ICER decreased, the incremental QALYs must increase (especially given that longer treatment increases costs). Therefore, the QALY difference in the JUPITER study should be even larger than the reported 0.31 when we apply our assumption of lifetime treatment.

Over a lifetime, the model estimated similar frequencies of revascularizations across strategies. Subsequently, differences in costs decreased, and anatomic approaches had significantly higher QALYs compared with functional testing (0.46 and 0.48 additional QALY gain for CTA with FFR_CT_ and CTA alone, respectively) and thus were cost-effective compared with functional testing. This principal finding was consistent across subgroups and sensitivity analyses and was further supported by the quasi-PSA. In all comparisons, anatomic approaches either dominated functional testing and/or were cost-effective, with cost per QALY below $50 000, making it high value according to the ACC/AHA.^41^ These results compare favorably with established strategies, such as lung cancer screening ($130 000/QALY)^58^ or screening for CAD in patients with type 2 diabetes or HIV.^59,60^ Moreover, assuming a willingness-to-pay threshold of $100 000/QALY, the probabilities that coronary CTA strategy and CTA with FFR_CT_ were cost-effective compared with functional testing is 69.4% and 65.4%, respectively. Additionally, our results are consistent with prior cost-effectiveness analysis publications, in which anatomical testing was shown to be cost-effective compared with functional assessment among those with low to intermediate pretest probability, thus, among patients with identical risk profiles as the PROMISE population.^46,61^ Nevertheless, using ICA-defined anatomical stenosis as a criterion standard puts noninvasive anatomical testing in a superior position; hence, further studies with invasive FFR as a criterion standard are warranted.^62^

A strength of our analysis is that the model was informed by individual patient demographic characteristics, CV risk factors, and CAD status from the PROMISE trial,^5^ which enrolled 10 003 patients at 192 US sites. Therefore, PROMISE is representative of the low-risk chest pain population and use of index testing, making the model results generalizable. Because we could accurately reproduce the patient management and clinical outcomes observed in PROMISE after 60 days and 2 years, our model appears to exactly simulate real-life clinical decision-making, including costs and outcomes for the coronary CTA only and the functional testing strategies for the first 2 years after the initial test. The implementation of FFR_CT_ and everything that happened after 2 years was modeled. However, the validity of our long-term model is strengthened by relying on actual clinical decision-making instead of assumptions during the first 2 years. From a medical treatment perspective, only differences in statin treatment between CTA and functional testing were modeled, based on the fact that underlying CAD was known after coronary CTA but not after functional testing. An additional strength was comprehensive validation of the model with observed outcomes in the PROMISE trial,^5^ including an accurate estimation of the distribution of applied functional tests (eg, SPECT, echo, exercise treadmill test), test findings, ICA and revascularization rates, health care costs, and incident MACE rates. Our study thus represents a high-quality cost-effectiveness analysis, given that other published analyses limit validation to mortality^63^ or ASCVD event rate^64^ or do not include model validation but only calibration.^65,66,67,68,69^ A further strength is that our principal finding that anatomic approaches were cost-effective compared with functional testing was stable over a wide range of assumptions in clinical care and patient characteristics and in most PSAs.

### Limitations

Our study has limitations, although most of these similarly affect all 3 strategies, including assumptions on MACE risk based on CV risk factors and CAD; the effects of medical therapy, except statin therapy; benefits and risks of ICA, PCI, and CABG; and risk of MACE after a first event. Similarly, inherent limitations of diagnostic accuracy values are based on core laboratory test readings, which were the same for all tests and strategies and were similar to published data. Moreover, the main results of this cost-effectiveness analysis are supported by model validation for 60-day and 2-year outcomes with PROMISE real-life observations and by the stability of the results across several sensitivity analyses and subgroups. The generalizability of our results to countries other than the United States is limited, given the differences in the health care systems in general and the differences in management of patients with SCP, including costs and type of diagnostic testing. A further limitation is that FFR_CT_ cannot be performed in all patients, limiting this strategy to a subset of patients.

## Conclusions

The results of this study suggest that anatomic strategies may present a more favorable initial diagnostic option in the evaluation of low-risk SCP compared with functional testing. This study further supports the most recent ESC guidelines on the management of chronic chest pain syndrome.

## Notes

Supplement. **eAppendix. **Supplementary Methods **eTable 1.** Accuracy of Noninvasive Diagnostic Tests **eTable 2.** Strata of Diagnostic Test Results **eTable 3.** Appropriate Treatment per AHA/ACC Guidelines **eTable 4.** Morbidity and Mortality **eTable 5. **Cost of Diagnostic Testing and Intervention **eTable 6.** Markov Microsimulation Model Validation: Comparison of Test Distribution and Findings, Interventions at 60 Days, Costs at 90 Days, and Health Outcomes at 2 Years Between Observed and Modeled Strategies in the PROMISE Trial **eTable 7.** Cost, QALYs, Incremental Cost-effectiveness Ratio and Life-Years Gained of Coronary CTA and Coronary CTA With FFR_CT_ Compared With Functional Testing, Stratified by Sex and Median Age of 60 Years **eFigure 1.** Modeling the Progression of CAD Using a Simulated Annealing Approach **eFigure 2.** Rate of ICA, Revascularization and Revascularization-to-ICA Ratio Based on Functional Strategy, Coronary CTA Strategy, and CTA With FFR_CT_ Strategy **eFigure 3.** Incremental Cost-effectiveness Plot and Cost-effectiveness Acceptability Curve for Functional Testing vs Coronary CTA and Functional Testing vs Coronary CTA With FFR_CT_ **eReferences.**
