# Supplementary material for: Cost-effectiveness Analysis of Anatomic vs Functional Index Testing in Patients With Low-Risk Stable Chest Pain
Source: JAMA Netw Open. 2020 Dec 14;3(12):e2028312. doi: 10.1001/jamanetworkopen.2020.28312 (PMC7737090; doi:10.1001/jamanetworkopen.2020.28312)
Supplement: Supplement. — eAppendix. Supplementary Methods eTable 1. Accuracy of Noninvasive Diagnostic Tests eTable 2. Strata of Diagnostic Test Results eTable 3. Appropriate Treatment per AHA/ACC Guidelines eTable 4. Morbidity and Mortality eTable 5. Cost of Diagnostic Testing and Intervention eTable 6. Markov Microsimulation Model Validation: Comparison of Test Distribution and Findings, Interventions at 60 Days, Costs at 90 Days, and Health Outcomes at 2 Years Between Observed and Modeled Strategies in the PROMISE Trial eTable 7. Cost, QALYs, Incremental Cost-effectiveness Ratio and Life-Years Gained of Coronary CTA and Coronary CTA With FFRCT Compared With Functional Testing, Stratified by Sex and Median Age of 60 Years eFigure 1. Modeling the Progression of CAD Using a Simulated Annealing Approach eFigure 2. Rate of ICA, Revascularization and Revascularization-to-ICA Ratio Based on Functional Strategy, Coronary CTA Strategy, and CTA With FFRCT Strategy eFigure 3. Incremental Cost-effectiveness Plot and Cost-effectiveness Acceptability Curve for Functional Testing vs Coronary CTA and Functional Testing vs Coronary CTA With FFRCT eReferences. [file jamanetwopen-e2028312-s001.pdf]

Supplemental Online Content

Karády J, Mayrhofer T, Ivanov A, et al. Cost-effectiveness analysis of anatomic vs functional index testing in patients with low-risk stable chest pain. *JAMA Netw Open*. 2020;3(12):e2028312. doi:10.1001/jamanetworkopen.2020.28312

eAppendix. Supplementary Methods
eTable 1. Accuracy of Noninvasive Diagnostic Tests
eTable 2. Strata of Diagnostic Test Results
eTable 3. Appropriate Treatment per AHA/ACC Guidelines
eTable 4. Morbidity and Mortality
eTable 5. Cost of Diagnostic Testing and Intervention
eTable 6. Markov Microsimulation Model Validation: Comparison of Test Distribution and Findings, Interventions at 60 Days, Costs at 90 Days, and Health Outcomes at 2 Years Between Observed and Modeled Strategies in the PROMISE Trial
eTable 7. Cost, QALYs, Incremental Cost-effectiveness Ratio and Life-Years Gained of Coronary CTA and Coronary CTA With FFR_{CT} Compared With Functional Testing, Stratified by Sex and Median Age of 60 Years
eFigure 1. Modeling the Progression of CAD Using a Simulated Annealing Approach
eFigure 2. Rate of ICA, Revascularization and Revascularization-to-ICA Ratio Based on Functional Strategy, Coronary CTA Strategy, and CTA With FFR_{CT} Strategy
eFigure 3. Incremental Cost-effectiveness Plot and Cost-effectiveness Acceptability Curve for Functional Testing vs Coronary CTA and Functional Testing vs Coronary CTA With FFR_{CT}
eReferences.

This supplemental material has been provided by the authors to give readers additional information about their work.

© 2020 Karády J et al. *JAMA Network Open*.

eAppendix. Supplementary Methods

1. ***Baseline model assumptions: The key drivers of decision making***
   1) The true underlying CAD status defined based on PROMISE data defines the health status (no CAD/non-obstructive CAD/obstructive CAD) of each individual entering the model.
   2) The index diagnostic tests (coronary CTA, functional testing, and coronary CTA+FFR_{CT}) are modeled to identify the true underlying CAD status with a diagnostic accuracy reported in the ESC Guidelines[1]
   3) Preventive treatment is simulated for those above 7.5% ASCVD risk score and for those with diagnosed CAD.
   4) Patients are referred to ICA as per the following diagnostic test findings:
      a. Functional testing: reversible myocardial ischemia (*supplemental table 2*);
      b. Coronary CTA: 70% in more than one vessel or 50% CAD in left main; 30-69% luminal stenosis CAD based on individual cardiovascular risk evaluation (*supplemental table 2*);
      c. CTA+FFR_{CT}: 70% in more than one vessel or 50% CAD in left main and 30-69% luminal stenosis CAD with an FFR_{CT} value <0.80.
   5) Patients are revascularized with the following invasive diagnostic findings:
      a. One- or two-vessel disease: PCI;
      b. Left main disease or three-vessel disease: CABG.
   6) MACE risk associated with a given health state (no CAD/non-obstructive CAD/obstructive CAD) is modeled based on age and sex specific cross-sectional data[2], which is modeled for each individual in monthly cycles until the end of life taking into account the potential change in health status. All-cause death risk is modeled in each monthly cycle for each patient based on US life tables.
   7) Treatment effects:
   8) Cost of FFR_{CT} CMS ($1450).

2. ***Underlying CAD status:*** the underlying disease status for patients of the CTA arm was derived from the CT core laboratory reads and ICA results. For patients of the CT arm but without this information available and for patients allocated to the functional testing arm, therefore patients without anatomical information on the underlying disease status, we assumed that the underlying prevalence and extent of CAD was similar compared to the anatomical testing arm, because of the randomized controlled trial design. Hence, to derive CAD status for those without this information available, we used a multivariable ordered logistic regression approach as imputation method. First, we applied multivariable ordered logistic regression to patients with available underlying disease status including various risk factors and demographics. Then based on the resulting regression parameters we predicted the underlying disease status in patients without known CAD status.

3. ***Progression of CAD*** is modeled as a function of age, gender, disease status and National Cholesterol Education Program (NCEP) risk score from a cohort of stable chest pain patients using a simulated annealing approach.[1, 2] In the model, each cycle started with the cohort divided among a set of mutually exclusive health states, where health states refer to a specific underlying CAD status. In each different health state, we modeled the events and progression that could occur within a cycle. Across a lifetime, transition to different health states was modeled in monthly cycles. From cycle to cycle the CAD status of a patient could remain the same or progress, and patients could suffer myocardial infarctions or could die from either cardiovascular disease or other causes. Therefore, the model was likely to start each cycle with a different distribution among those health states.

4. ***Definitions of cost, QALY, cost-effectiveness analysis, ICER and life years gained.***

   Cost: direct medical costs such as cost for diagnostic testing, interventions, and subsequent medication associated with each clinical strategy in US.

   QALYs: measures quality adjusted life years (QALYs) by combining the quality and the quantity of life lived: the time spent in a particular health state measured in years multiplied by an utility weight ranging from 0 to 1, where 1 reflects perfect health and 0 the worst health state (in this study the worst health state is assumed to be death).

   Cost-effectiveness analysis: estimates the costs (in this study measured in US$) and health gains (in this study measured in QALYs) of alternative strategies. A strategy is called dominant compared to another strategies if is both, cost saving and more effective (in this study this equals a higher number of QALYs). In general, 'new'

© 2020 Karády J et al. *JAMA Network Open.*

strategies are more effective than 'old' strategies but at higher cost. In this case, a strategy is called cost effective if the cost per additional QALY, i.e. the ICER, is below $100,000 (see also next ICER paragraph).

ICER: measures the incremental cost-effectiveness ratio (ICER). It is defined by the cost difference between two interventions divided by the difference in healthcare effect and reflects the additional (incremental) cost associated with one unit increase of the measured healthcare effect (here QALYs).

$$ICER = \frac{Cost_{new\ strategy} - Cost_{alternative\ strategy}}{QALYs_{new\ strategy} - QALYs_{alternative\ strategy}}$$

When calculating the ICER, each strategy is compared with the next best alternative, based on the economic concept of opportunity costs. In this study the ICER shows the additional cost per QALY gained. We consider a diagnostic strategy *cost-effective*, when the ICER is below the cost-effectiveness threshold of 100,000$/QALY. A strategy is *cost-saving*, if additional QALY is gained at a lower cost compared to the other strategy.

Life years gained: expresses the additional number of years of life that a person lives as a result of receiving a certain diagnostic strategy.

© 2020 Karády J et al. *JAMA Network Open.*

eTables

eTable 1. Accuracy of Noninvasive Diagnostic Tests

	Diagnosis of CAD	
**Diagnostic test**	**Sensitivity (%)**	**Specificity (%)**
Exercise ECG[3]*	45-50	85-90
Exercise STECHO [3]*	80-85	80-88
Dobutamine STECHO [3]*	79-83	82-86
Exercise stress SPECT[3]*	73-92	63-87
Vasodilator stress SPECT[3]*	90-91	75-84
Coronary CTA[3]*	95-99	64-83
FFR_{CT}[3, 4†]	75-89	82-89
Per Vessel FFR_{CT}[4†]	75-89	82-89
Per Patient FFR_{CT}[4†]	77-92	72-84

* Gold standard invasive coronary angiography. † Gold standard invasive FFR. CTA=Coronary CT angiography; Echo=Echocardiography; ECG=Electrocardiogram; FFR=Fractional Flow reserve; FFR_{CT}=Non-invasive fractional flow reserve derived from computed tomography; STECHO=Stress echocardiography; SPECT=Single photon emission computed tomography

© 2020 Karády J et al. *JAMA Network Open.*

eTable 2. Strata of Diagnostic Test Results

<table>
<tr><th colspan="2">Anatomical testing</th><th colspan="4">Functional testing</th></tr>
<tr><th>Test strata</th><th>CTA</th><th>Test strata</th><th>ETT</th><th>SPECT</th><th>STECHO</th></tr>
<tr><td>Severely abnormal</td><td>≥70% stenosis ≥2 vessel disease OR ≥50% LM stenosis</td><td>Severely abnormal</td><td>Ischemic ECG ST changes consistent with ischemia +either severe ventricular arrhythmia OR hypotension</td><td>Large territory inducible Ischemia or mixed defect Septal/anterior/apical territory or other single territory with transient ischemic dilatation or 2 or more coronary territories with ischemia</td><td>Large territory inducible Ischemia or mixed defect Wall motion abnormality or mixed abnormality (infarct and ischemia) Isolated Septal/anterior/apical or other single territory during stress or
2 or more coronary territories</td></tr>
<tr><td>Moderately abnormal</td><td>30-69% stenosis in one major Vessels / branch</td><td rowspan="2">Mildly abnormal</td><td rowspan="2">Early positive TM
Failure to reach stage 2 (<3:00 min) with ST changes OR symptoms reproduced OR any arrhythmia or hypotension OR
Late positive TM
More than stage 2 (>3:00 min) but failure to finish protocol or target heart rate achieved due to ST changes OR symptoms reproduced
OR any arrhythmia or hypotension</td><td rowspan="2">Inducible Ischemia or mixed defect
Perfusion abnormality in one coronary territory (Lateral or Inferior/posterior)
OR Normal imaging but Early positive TM
Failure to reach stage 2 (<3:00 min) with ST changes OR symptoms reproduced
OR any arrhythmia or hypotension
OR Positive ECG
Normal perfusion or fixed perfusion defect (Scar) OR Normal imaging but Late positive TM
More than stage 2 (>3:00 min) but failure to finish protocol or target heart rate achieved due to ST changes
OR symptoms reproduced
OR any arrhythmia or hypotension</td><td rowspan="2">Inducible Ischemia or mixed defect Wall motion abnormality or mixed abnormality (infarct and ischemia) in one coronary territory (Lateral or Inferior/posterior)
OR Normal imaging but Early positive TM
Failure to reach stage 2 (<3:00 min) with ST changes OR symptoms reproduced or any arrhythmia or hypotension OR Positive ECG but normal wall motion or resting wall motion abnormality without inducible ischemia
OR Normal imaging but Late positive TM
More than stage 2 (>3:00 min) but failure to finish protocol or target heart rate achieved due to ST changes OR symptoms reproduced OR any arrhythmia or hypotension</td></tr>
<tr><td>Mildly abnormal</td><td>1-29% stenosis in any major vessels / branch OR <50% left main stenosis</td></tr>
<tr><td>Normal</td><td>Absence of coronary atherosclerosis</td><td>Normal</td><td>Normal ECG, absence of symptoms during exercise, and normal exercise duration</td><td>Normal ECG, absence of symptoms during exercise, normal exercise duration, and normal imaging (absence of any findings suggesting myocardial abnormalities including fixed perfusion defects)</td><td>Normal ECG, absence of symptoms during exercise, normal exercise duration, and normal imaging (absence of any findings suggesting myocardial abnormalities including fixed wall motion abnormalities)</td></tr>
</table>

Diagnostic strata defined as previously reported.[5] ECG=Electrocardiogram; LM=Left main coronary artery; STECHO=Stress echocardiography; SPECT=Single photon emission computed tomography; TM=Treadmill test.

© 2020 Karády J et al. *JAMA Network Open.*

eTable 3. Appropriate Treatment per AHA/ACC Guidelines

**Diagnosis**		**Treatment**	
MI	CAD	Intervention	Medical treatment
Yes	Non-obstructive CAD	None	Aspirin, high-dose Statin, Clopidogrel (one year only), Beta blocker, and ACE
Yes	1 or 2 VD	PCI	Aspirin, high-dose Statin, Clopidogrel (one year only), Beta blocker, and ACE
Yes	3 VD or LM disease	CABG	Aspirin, high-dose Statin, Clopidogrel (one year only), Beta blocker, and ACE
No	No CAD	None	Aspirin and Statin (Primary Prevention only for patients meeting appropriate risk threshold based on risk factors)
No	Non-obstructive CAD	None	Aspirin and Statin
No	1 or 2 VD	None	Aspirin, Statin, and betablocker and possibly ACE inhibitors if diabetes or hypertension
No	3 VD or LM disease	CABG	Aspirin, Statin, and Betablocker and possibly ACE inhibitor if diabetes or hypertension

MI=Myocardial Infarction; CABG=Coronary Artery Bypass Graft; CAD=Coronary Artery Disease; LM disease=Left-Main disease; PCI=Percutaneous Coronary Intervention; VD=Vessel Disease.

© 2020 Karády J et al. *JAMA Network Open.*

eTable 4. Morbidity and Mortality

Variable	Value	Source
**Periprocedural mortality risk for diagnostic tests and interventions (30-day mortality risks in percentages)**		
CABG	1.0	[6-8]
PCI	0.5	[9]
Diagnostic invasive angiography	0.1	[10]
Coronary CT Angiography	0.000006	[11]
Stress test	0.00005	[12]
**Disease specific increase in mortality risk (hazard ratios)**		
Non-obstructive CAD (vs no CAD)	3.16	[13]
Obstructive one-vessel disease (vs no CAD)	5.78	[13]
Obstructive two-vessel disease (vs no CAD)	6.65	[13]
Obstructive three-vessel disease / left-main disease (vs no CAD)	8.48	[13]
**Treatment effect (mortality decrease) (hazard ratios)**		
Obstructive CAD	0.77	[14]
**Additional mortality risks for ACS (first year)**		
Non-ST segment myocardial infarction		
Patient with obstructive CAD	0.046	[15, 16]
Patient with non-obstructive or no CAD	0.014	[17]
Unstable Angina		
Patients with CAD	0.029	[15, 16]
Patient with non-obstructive or no CAD	0.014	[17]
**Mortality risk increase for missed ACS (30-day) (hazard ratio)**		
Non-ST segment myocardial infarction	1.90	[18, 19]
Unstable Angina	1.70	[18, 19]
**Monthly incidence of recurrent events (myocardial infarction)**		
No CAD	0.000067	[20]
Non-obstructive CAD	0.000281	[20]
Obstructive CAD	0.001063	[20]

ACS=Acute coronary syndrome; CABG=Coronary artery bypass graft; CAD=Coronary artery disease; ICA=Invasive coronary angiography; PCI=Percutaneous coronary intervention.

© 2020 Karády J et al. *JAMA Network Open.*

eTable 5. Cost of Diagnostic Testing and Intervention

Test as in Simulation	Test as in PROMISE	N	Cost	Weighted Average Cost	Source
**Diagnostic testing**					
SPECT	Pharm MPI	3,903	1,132		PROMISE[21]
	Exercise MPI	2,396	946	$1,061	PROMISE[21]
STECHO	Pharm Echo	152	501		PROMISE[21]
ETT	Exercise Echo	632	514	$511	PROMISE[21]
	Exercise EKG	455	174	$174	PROMISE[21]
CTA	CTA	489	404	$404	PROMISE[21]
FFR_{CT}			1,450	$1,450	CMS.gov[22]
ICA	ICA	172	3,656	$3,656	PROMISE[21]
**Intervention**					
PCI	PCI	325	12,779	$12,779	PROMISE[21]
CABG	CABG	37	32,546	$32,546	PROMISE[21]

CABG=Coronary artery bypass graft; CTA=Computed tomography angiography; ECG=Electrocardiogram; ETT=Exercise tolerance test; FFR_{CT}=Non-invasive fractional flow reserve derived from computed tomography; ICA=Invasive coronary angiography; MPI=Myocardial perfusion imaging; PCI=Percutaneous coronary intervention; Pharm=Pharmacological; STECHO=Stress echocardiography; SPECT=Single photon emission computed tomography.

© 2020 Karády J et al. *JAMA Network Open.*

eTable 6. Markov Microsimulation Model Validation: Comparison of Test Distribution and Findings, Interventions at 60 Days, Costs at 90 Days, and Health Outcomes at 2 Years Between Observed and Modeled Strategies in the PROMISE Trial

	**CTA Strategy**		**Functional Strategy**	
**Index test**	**Observed Results***	**Simulated Results**	**Observed Results***	**Simulated Results**
	**N=4,996**	**N=499,600†**	**N=5,007**	**N=500,700†**
**60 days follow-up**				
**Initial diagnostic test finding‡**				
Normal (%)	33.4	30.0	78.0	70.4
Mildly abnormal (%)	29.0	34.2	14.1	20.8
Moderately abnormal (%)	31.6	31.4		
Severely abnormal (%)	5.9	4.4	7.9	8.8
**Non-invasive diagnostic testing**				
CTA (%)	100.0	100.0	0.0	0.0
FFR_{CT} (%)	0.0	0.0	0.0	0.0
SPECT (%)	1.8	1.8	67.5	67.2
Stress ECHO (%)	0.0	0.0	22.4	22.5
ETT (%)	0.0	0.0	10.2	10.4
**Invasive diagnostic testing and Interventions**				
ICA (%)	12.2	12.3	8.1	8.2
Coronary Revascularization	6.2	6.4	3.2	3.3
PCI (%)	4.7	4.6	2.5	2.6
CABG (%)	1.5	1.9	0.7	0.7
**Cost**				
Cost in US $ (at 90 days)	$2,494[21]	$2,546	$2,240[21]	$2,189
**2 years follow-up**				
**Composite MACE** - Death or nonfatal MI (%)	2.1	2.3	2.2	2.4
All cause death (%)	1.5	1.6	1.5	1.7
Nonfatal MI (%)	0.6	0.7	0.8	0.8
CV Death or nonfatal MI (%)	1.4	1.3	1.7	1.4

*Observed results are from the PROMISE trial.[21, 23] † Each patient 100 times. ‡ Diagnostic test finding strata: *supplement table 2*.

CABG=Coronary artery bypass graft; CTA=Computed tomography angiography; CV=Cardiovascular; ECG=Electrocardiogram; ETT=Exercise tolerance test; FFRCT=Non-invasive fractional flow reserve derived from computed tomography; ICA=Invasive coronary angiography; PCI=Percutaneous coronary intervention; MACE=Major adverse cardiovascular event; MI=Myocardial infarction; MPI=Myocardial perfusion imaging; SECHO=Stress echocardiography; SPECT = Single photon emission computed tomography; US=United States.

eTable 7. Cost, QALYs, Incremental Cost-effectiveness Ratio and Life-Years Gained of Coronary CTA and Coronary CTA With FFR_{CT} Compared With Functional Testing, Stratified by Sex and Median Age of 60 Years

	Undiscounted cost	Cost difference[a]	Undiscounted QALYs	QALY difference[a]	Discounted ICER ($/QALY)[b]	Life years gained (years)
**Coronary CTA vs Functional Testing**						
**Males**						
Functional strategy	$ 8,652		24.27			26.19
Coronary CTA strategy	$ 9,620	$968	24.77	0.50	$ 3,559 / QALY[c]	26.74
**Females**						
Functional strategy	$ 7,393		25.05			26.79
Coronary CTA strategy	$ 7,841	$448	25.51	0.46	$ 1,912 / QALY[c]	27.29
**Below 60 years of age**						
Functional strategy	$ 8,427		27.15			29.15
Coronary CTA strategy	$ 8,990	$563	27.59	0.44	$ 2,616 / QALY[c]	29.64
**Above 60 years of age**						
Functional strategy	$ 7,549		22.20			23.85
Coronary CTA strategy	$ 8,374	$825	22.72	0.52	$ 2,842 / QALY[c]	24.41
**Coronary CTA+FFR_{CT} vs Functional Testing**						
**Males**						
Functional strategy	$ 8,652		24.27			26.19
CTA+FFR_{CT} strategy	$ 8,147	-$505	24.75	0.48	$ 192 / QALY[§]	26.72
**Females**						
Functional strategy	$ 7,393		25.05			26.79
CTA+FFR_{CT} strategy	$ 6,391	-$1,002	25.49	0.45	CTA+FFR_{CT} dominates[d] Functional Testing	27.27
**Below 60 years of age**						
Functional strategy	$ 8,427		27.15			29.15
CTA+FFR_{CT} strategy	$ 7,395	-$1,032	27.57	0.42	CTA+FFR_{CT} dominates[d] Functional Testing	29.62
**Above 60 years of age**						
Functional strategy	$ 7,549		22.20			23.85
CTA+FFR_{CT} strategy	$ 7,048	-$501	22.70	0.51	CTA+FFR_{CT} dominates Functional Testing	24.39

[a]Cost and QALY differences are expressed in reference to functional strategy.
[b]Discounted at 3% annually as recommended by the US Panel on Cost-Effectiveness in Health and Medicine.[38,39]
[c]A strategy is considered cost-effective when ICER is below $100,000/QALY.[32]
[d]A strategy dominated the other, if it has lower cost and higher QALY compared to the comparator strategy.
Abbreviations: CTA, Computed tomography angiography; FFR_{CT}, Non-invasive fractional flow reserve derived from computed tomography; ICER, Incremental cost-effectiveness ratio; ICA, Invasive coronary angiography; MI, Myocardial infarction; QALY, Quality adjusted life years.

© 2020 Karády J et al. *JAMA Network Open.*

eFigures

eFigure 1. Modeling the Progression of CAD Using a Simulated Annealing Approach

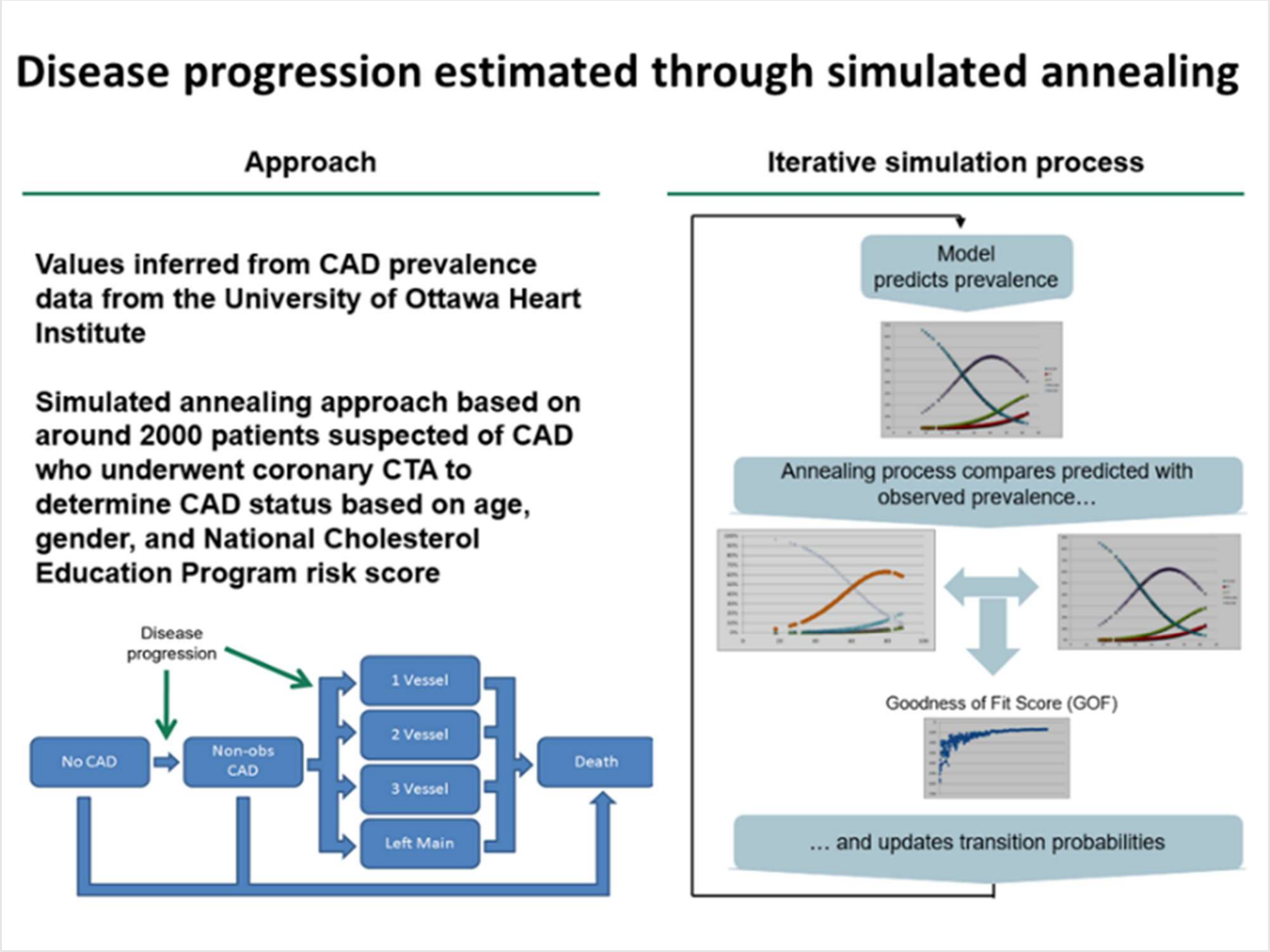

CAD=Coronary artery disease.

eFigure 2. Rate of ICA, Revascularization and Revascularization-to-ICA Ratio Based on Functional Strategy, Coronary CTA Strategy, and CTA With FFR_{CT} Strategy

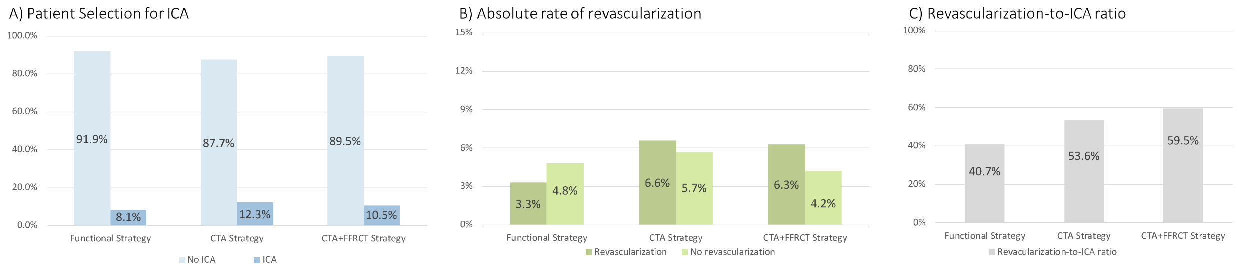

Panel A: Rate of ICA based on coronary functional strategy, CTA strategy, and CTA+FFR_{CT} strategy. Panel B: Rate of revascularization when ICA was performed based on the three strategies. Panel C: the yield of ICA per the three strategies.

Abbreviations: CTA, CT angiography; ICA, Invasive coronary angiography; FFR_{CT}, Non-invasive fractional flow reserve derived from computed tomography; ICA, Invasive coronary angiography.

© 2020 Karády J et al. *JAMA Network Open.*

eFigure 3. Incremental Cost-effectiveness Plot and Cost-effectiveness Acceptability Curve for Functional Testing vs Coronary CTA and Functional Testing vs Coronary CTA With FFR_{CT}

A) Coronary CTA vs Functional testing

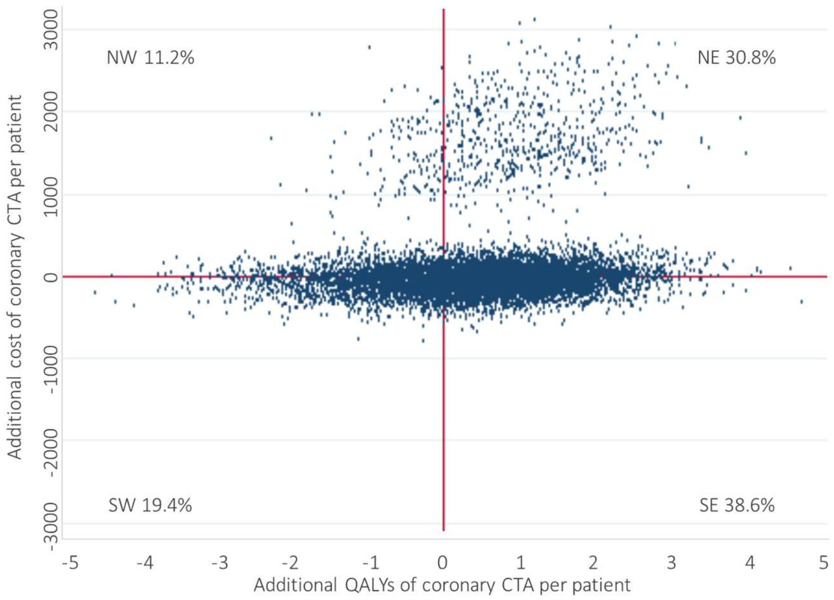

B) Coronary CTA+FFR_{CT} vs Functional testing

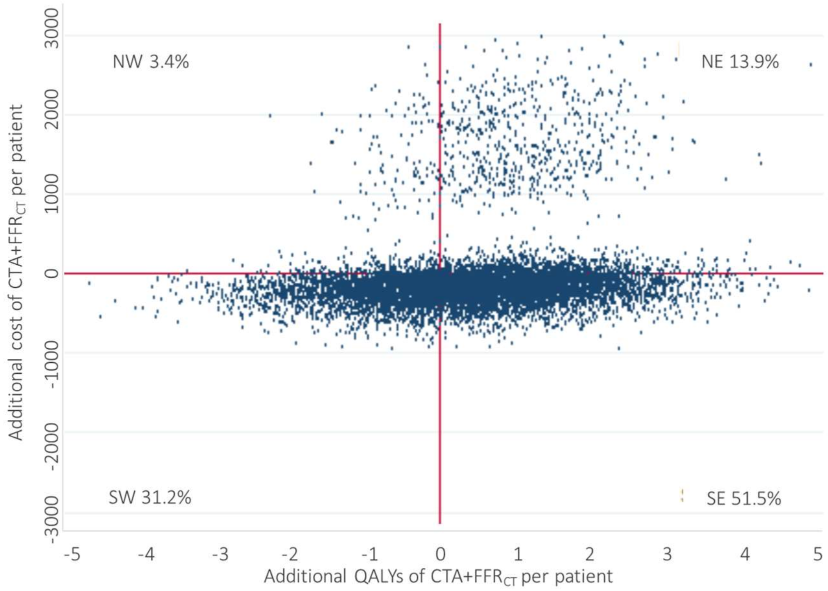

© 2020 Karády J et al. *JAMA Network Open.*

C) Coronary CTA vs Functional testing

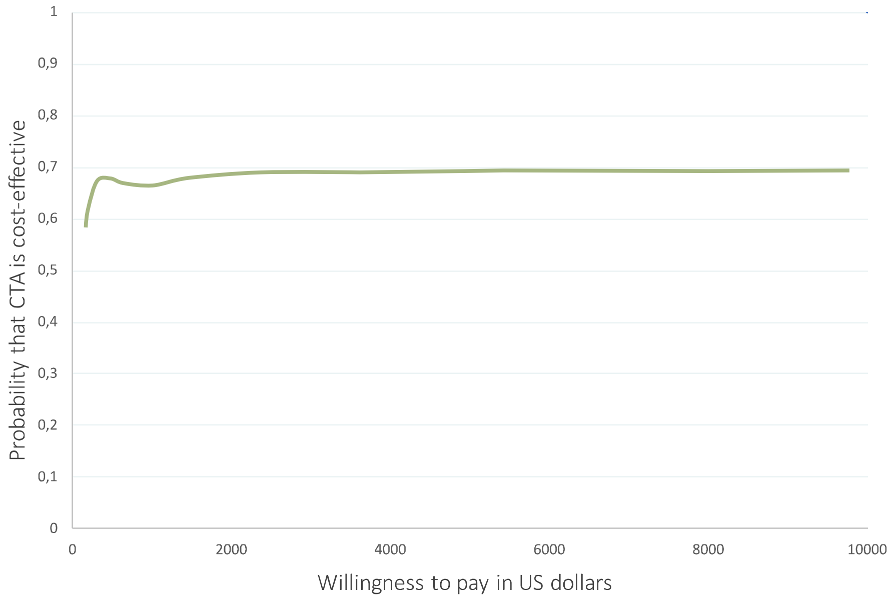

D) Coronary CTA+FFR_{CT} vs Functional testing

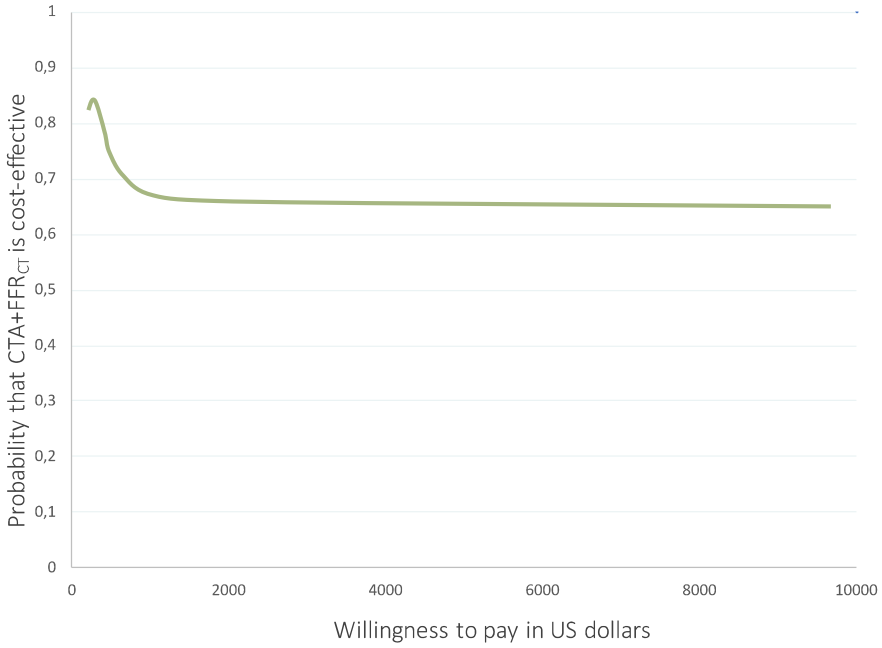

Panel (A) and *(B)* show the possible outcomes in effectiveness as measured in additional QALYs and costs given the uncertainty in the input data. Each outcome is expressed in incremental costs and incremental effects (i.e. the difference in costs and effects for *(A)* coronary CTA and *(B)* coronary CTA+FFR_{CT} as compared to functional testing).

© 2020 Karády J et al. *JAMA Network Open.*

The majority of the simulation outcomes are in the south of the origin indicating that both coronary CTA *(A)* and coronary CTA+FFR_{CT} *(B)* are for the majority of cases less costly compared to functional testing. Moreover, simulation outcomes located east from the origin, indicate a higher effectiveness (measured in QALYs) for both coronary CTA *(A)* and coronary CTA+FFR_{CT} *(B)*. As indicated in the southeast sector of panel (A) and (B), coronary CTA alone and coronary CTA+FFR_{CT} dominated functional testing in 38.6% and 51.5% of the cases, respectively, since they rendered to be less costly and more effective.

In *panel (C)* and *(D)*, cost-effectiveness acceptability curves show the likelihood that *(C)* coronary CTA alone and *(D)* coronary CTA+FFR_{CT} are cost-effective compared to functional testing given different willingness-to-pay thresholds per QALY. The curves show the willingness to pay up to $10,000 per QALY because the likelihood does not change beyond that threshold. The common threshold of $100,000/QALY would render CTA in 69.4% and CTA+FFR_{CT} in 65.4% of the cases as cost-effective as compared to functional testing.

© 2020 Karády J et al. *JAMA Network Open.*

eReferences

1. Task Force M, Montalescot G, Sechtem U, Achenbach S, Andreotti F, Arden C, Budaj A, Bugiardini R, Crea F, Cuisset T, et al. 2013 ESC guidelines on the management of stable coronary artery disease: the Task Force on the management of stable coronary artery disease of the European Society of Cardiology. *Eur Heart J*. 2013;34:2949-3003.
2. Chow BJ, Small G, Yam Y, Chen L, Achenbach S, Al-Mallah M, Berman DS, Budoff MJ, Cademartiri F, Callister TQ, et al. Incremental prognostic value of cardiac computed tomography in coronary artery disease using CONFIRM: COroNary computed tomography angiography evaluation for clinical outcomes: an InteRnational Multicenter registry. *Circ Cardiovasc Imaging*. 2011;4:463-72.
3. Kong CY, McMahon PM and Gazelle GS. Calibration of disease simulation model using an engineering approach. *Value Health*. 2009;12:521-9.
4. Norgaard BL, Leipsic J, Gaur S, Seneviratne S, Ko BS, Ito H, Jensen JM, Mauri L, De Bruyne B, Bezerra H, et al. Diagnostic performance of noninvasive fractional flow reserve derived from coronary computed tomography angiography in suspected coronary artery disease: the NXT trial (Analysis of Coronary Blood Flow Using CT Angiography: Next Steps). *J Am Coll Cardiol*. 2014;63:1145-1155.
5. Hoffmann U, Ferencik M, Udelson JE, Picard MH, Truong QA, Patel MR, Huang M, Pencina M, Mark DB, Heitner JF, et al. Prognostic Value of Noninvasive Cardiovascular Testing in Patients With Stable Chest Pain: Insights From the PROMISE Trial (Prospective Multicenter Imaging Study for Evaluation of Chest Pain). *Circulation*. 2017;135:2320-2332.
6. Peterson ED, Coombs LP, DeLong ER, Haan CK and Ferguson TB. Procedural volume as a marker of quality for CABG surgery. *JAMA*. 2004;291:195-201.
7. Birkmeyer JD, Siewers AE, Finlayson EV, Stukel TA, Lucas FL, Batista I, Welch HG and Wennberg DE. Hospital volume and surgical mortality in the United States. *N Engl J Med*. 2002;346:1128-37.
8. Hannan EL, Racz MJ, Walford G, Jones RH, Ryan TJ, Bennett E, Culliford AT, Isom OW, Gold JP and Rose EA. Long-term outcomes of coronary-artery bypass grafting versus stent implantation. *N Engl J Med*. 2005;352:2174-83.
9. Anderson HV, Shaw RE, Brindis RG, Hewitt K, Krone RJ, Block PC, McKay CR and Weintraub WS. A contemporary overview of percutaneous coronary interventions. The American College of Cardiology-National Cardiovascular Data Registry (ACC-NCDR). *J Am Coll Cardiol*. 2002;39:1096-103.
10. Johnson LW, Lozner EC, Johnson S, Krone R, Pichard AD, Vetrovec GW and Noto TJ. Coronary arteriography 1984-1987: a report of the Registry of the Society for Cardiac Angiography and Interventions. I. Results and complications. *Cathet Cardiovasc Diagn*. 1989;17:5-10.
11. Morcos SK. Review article: Acute serious and fatal reactions to contrast media: our current understanding. *Br J Radiol*. 2005;78:686-93.
12. Weinberg L, Kandasamy K, Evans SJ and Mathew J. Fatal cardiac rupture during stress exercise testing: case series and review of the literature. *South Med J*. 2003;96:1151-3.
13. Cho I, Al'Aref SJ, Berger A, B OH, Gransar H, Valenti V, Lin FY, Achenbach S, Berman DS, Budoff MJ, et al. Prognostic value of coronary computed tomographic angiography findings in asymptomatic individuals: a 6-year follow-up from the prospective multicentre international CONFIRM study. *Eur Heart J*. 2018;39:934-941.
14. Pope JH, Aufderheide TP, Ruthazer R, Woolard RH, Feldman JA, Beshansky JR, Griffith JL and Selker HP. Missed diagnoses of acute cardiac ischemia in the emergency department. *N Engl J Med*. 2000;342:1163-70.
15. Armstrong PW, Fu Y, Chang WC, Topol EJ, Granger CB, Betriu A, Van de Werf F, Lee KL and Califf RM. Acute coronary syndromes in the GUSTO-IIb trial: prognostic insights and impact of recurrent ischemia. The GUSTO-IIb Investigators. *Circulation*. 1998;98:1860-8.
16. Roe MT, Harrington RA, Prosper DM, Pieper KS, Bhatt DL, Lincoff AM, Simoons ML, Akkerhuis M, Ohman EM, Kitt MM, et al. Clinical and therapeutic profile of patients presenting with acute coronary syndromes who do not have significant coronary artery disease.The Platelet Glycoprotein IIb/IIIa in Unstable Angina: Receptor Suppression Using Integrilin Therapy (PURSUIT) Trial Investigators. *Circulation*. 2000;102:1101-6.
17. Arias E. United States life tables, 2010. *Natl Vital Stat Rep*. 2014;63:1-63.
18. Chaitman BR, Bourassa MG, Davis K, Rogers WJ, Tyras DH, Berger R, Kennedy JW, Fisher L, Judkins MP, Mock MB, et al. Angiographic prevalence of high-risk coronary artery disease in patient subsets (CASS). *Circulation*. 1981;64:360-7.

© 2020 Karády J et al. *JAMA Network Open*.

19. Gandhi SK, Jensen MM, Fox KM, Smolen L, Olsson AG and Paulsson T. Cost-effectiveness of rosuvastatin in comparison with generic atorvastatin and simvastatin in a Swedish population at high risk of cardiovascular events. *Clinicoecon Outcomes Res*. 2012;4:1-11.
20. Stone GW, Ware JH, Bertrand ME, Lincoff AM, Moses JW, Ohman EM, White HD, Feit F, Colombo A, McLaurin BT, et al. Antithrombotic strategies in patients with acute coronary syndromes undergoing early invasive management: one-year results from the ACUITY trial. *JAMA*. 2007;298:2497-506.
21. Mark DB, Federspiel JJ, Cowper PA, Anstrom KJ, Hoffmann U, Patel MR, Davidson-Ray L, Daniels MR, Cooper LS, Knight JD, et al. Economic Outcomes With Anatomical Versus Functional Diagnostic Testing for Coronary Artery Disease. *Ann Intern Med*. 2016;165:94-102.
22. CMS. U.S. Centers for Medicare & Medicaid Services. 2017;2019.
23. Douglas PS, Hoffmann U, Patel MR, Mark DB, Al-Khalidi HR, Cavanaugh B, Cole J, Dolor RJ, Fordyce CB, Huang M, et al. Outcomes of anatomical versus functional testing for coronary artery disease. *N Engl J Med*. 2015;372:1291-300.
24. Siegel JE, Weinstein MC, Russell LB and Gold MR. Recommendations for reporting cost-effectiveness analyses. Panel on Cost-Effectiveness in Health and Medicine. *JAMA*. 1996;276:1339-41.
25. Weinstein MC, Siegel JE, Gold MR, Kamlet MS and Russell LB. Recommendations of the Panel on Cost-effectiveness in Health and Medicine. *JAMA*. 1996;276:1253-8.
26. Hunink M. G. M., Weinstein M. C., Wittenberg E., Drummond M. F., Pliskin J. S., Wong J. B. and B. GP. *Decision Making in Health and Medicine: Integrating Evidence and Values*: Cambridge University Press; 2014.

© 2020 Karády J et al. *JAMA Network Open.*
